# A Somatic Movement Approach to Fostering Emotional Resiliency through Laban Movement Analysis

**DOI:** 10.3389/fnhum.2017.00410

**Published:** 2017-09-07

**Authors:** Rachelle P. Tsachor, Tal Shafir

**Affiliations:** ^1^Department of Theatre, School of Theatre & Music, The University of Illinois at Chicago Chicago, IL, United States; ^2^The Graduate School of Creative Arts Therapies, The University of Haifa Haifa, Israel; ^3^The Emili Sagol Creative Arts Therapies Research Center, The University of Haifa Haifa, Israel

**Keywords:** Laban Movement Analysis, embodiment, bodily emotional expression, dance-movement therapy, body self-efficacy, body-mind, emotional resiliency, somatics

## Abstract

Although movement has long been recognized as expressing emotion and as an agent of change for emotional state, there was a dearth of scientific evidence specifying which aspects of movement influence specific emotions. The recent identification of clusters of Laban movement components which elicit and enhance the basic emotions of anger, fear, sadness and happiness indicates which types of movements can affect these emotions (Shafir et al., 2016), but not how best to apply this knowledge. This perspective paper lays out a conceptual groundwork for how to effectively use these new findings to support emotional resiliency through voluntary choice of one's posture and movements. We suggest that three theoretical principles from Laban Movement Analysis (LMA) can guide the gradual change in movement components in one's daily movements to somatically support shift in affective state: (A) Introduce new movement components in developmental order; (B) Use LMA affinities-among-components to guide the expansion of expressive movement range and (C) Sequence change among components based on Laban's Space Harmony theory to support the gradual integration of that new range. The methods postulated in this article have potential to foster resiliency and provide resources for self-efficacy by expanding our capacity to adapt emotionally to challenges through modulating our movement responses.

## Introduction: mechanisms for somatic movement's effects on emotions

The idea that certain postures and movements are associated with specific emotions is not new: The concept of expressing emotions through body language dates as far back as Aristotle (Lee, 2008). While it is widely accepted that emotions are expressed in movement and posture, evidence suggests that the connection between movement and emotion is bidirectional: that we can affect emotional state by changing posture and movement. This concept originates in Darwin's ideas (Darwin, 1882) and the James-Lang theory (James, 1884), which postulate that bodily responses to stimuli are necessary for emotional experience, and therefore feelings are not the causes of autonomic system activation and emotional behavior, but rather are the consequence of them.

Subsequent theorists in the field of emotion followed this idea, proposing that sensory feedback from facial and postural movements contributes significantly to emotional experience (Tomkins, 1962; Laird, 1974; Izard, 1993). In recent years, this theory has been re-formulated in neurophysiological terms by Antonio Damasio, who asserted that emotions are evoked by interoceptive and proprioceptive feedback from the body and our conscious feelings result from our perception of this somatic input (Damasio et al., 2000; Damasio and Carvalho, 2013). The uncovering of neuronal underpinnings of interoception (Craig, 2002; Critchley, 2005) and identification of the anterior insular cortex as the brain region in which representation of internal bodily states becomes available to conscious awareness (Craig, 2009; Harrison et al., 2010), provide plausible neurocircuits in support of this hypothesis (Critchley and Harrison, 2013). Evidence suggesting that the effects of facial expressions on affective state are attained through proprioception comes from mimicry and botulin toxin studies: Mimicry, which involves facial muscle contraction, more strongly activated brain emotional processing regions compared to mere observation of facial expression (Carr et al., 2003), and mimicry of emotional but not ingestive (chewing, licking) facial expressions activated brain emotional processing regions, where the magnitude of facial movement predicted responses within the right insula (Lee et al., 2006). Reduced muscle activation during emotional facial expressions following botulin toxin treatment and corresponding reduction in proprioceptive feedback, attenuated neural activation in the amygdala (Hennenlotter et al., 2009) and weakened emotional experience (Davis et al., 2010). Although fMRI data cannot be collected during whole-body emotionally expressive movements, observation of such movements has been shown to activate emotional processing regions (de Gelder et al., 2004; Pichon et al., 2008) and to modify affective state (Shafir et al., 2013). Based on the embodied simulation theory (Niedenthal et al., 2010; Gallese and Sinigaglia, 2011), movement-related brain activation during motor execution is similar to that during motor observation of the same movements. Thus, brain activation during motor observation may imply what happens during motor execution. Motor observation creates neuronal activity in the observer's motor system which is likely related to simulation of the observed movement (Borgomaneri et al., 2012), and sensory cortex activation during movement observation (Gazzola and Keysers, 2009) indicates that this simulation probably also includes simulation of the expected proprioceptive feedback from the simulated movement (Valchev et al., 2016), which probably elicits the feelings associated with those emotional movements (Bastiaansen et al., 2009). People tend to perceive certain movements as expressing specific emotions (Dael et al., 2012; Kleinsmith and Bianchi-Berthouze, 2013), which suggests the existence of associations in our brain between certain movements and specific emotions. Thus, similar to what happens during observation of bodily emotional expressions, the proprioception from emotional movements during execution of such expressions probably activates emotional processing regions to enhance the associated feelings.

Damasio's theory implies that by deliberately choosing our motor behavior, we can affect our feelings. This was stated by Riskind as early as 1984 (Riskind, 1984) and demonstrated by subsequent studies (for review, see Shafir, 2015). While these studies used specific, scripted movements to demonstrate the effects of movement on emotions, in life, people use a variety of movements to express their emotions. Accordingly, Shafir et al. (2016) investigated complex, non-scripted improvised movements, using Laban Movement Analysis (LMA) (a comprehensive method for describing and documenting human movement) and identified unique sets of Laban movement components which can elicit the feelings of happiness, sadness, fear, or anger. These findings concur with the motor components of scripted movements and postures studied in Duclos et al. (1989), Flack et al. (1999), Duclos and Laird (2001), Koch et al. (2007), Shafir et al. (2013) and other studies. While the associations between emotions and specific motor components have been mostly used in the past for diagnosis or for emotion recognition, we are the first, to the best of our knowledge, to suggest specific techniques for using these associations for emotion regulation.

## The potential of movement-based somatic techniques to foster emotional resiliency

Dance movement (psycho)therapy (DMT) has long relied upon movement to both facilitate emotional expression and change emotional state. Movement responses evoked by DMT have potential to help people cope more effectively with their external world (Smallwood, 1978) and they positively affect mood and wellbeing (Koch et al., 2014). One established DMT method, developed by Blanche Evan, stimulates emotional transformation and psychological growth by teaching new movement qualities to expand movement vocabulary, and by exploring, through movement improvisation, personally meaningful images, ideas, statements, or new responses to difficult situations, to affect emotional state and increase resiliency (Bernstein, 1995). Evan's interventions are based on her psychophysical concept that (1) all experiences occur concurrently on psychological and physical (bodily) levels; and (2) the body impacts the psyche as the psyche impacts the body. This psychophysical concept is consistent with Damasio's theories. The goal underlying DMT movement suggestions is to facilitate moving in new ways not regularly used, to support emotional shift.

If feedback from movement affects emotional state, then reducing the frequency of Laban motor components shown to enhance unwanted emotional state may also empower emotional resiliency. Established somatic movement techniques for moment-to-moment movement awareness, such as the Feldenkrais and Alexander Techniques, empower self-modification of daily movement, and thus could be used to affect emotional state, for example, by learning to avoid a slouched posture through Alexander Technique. Increased body awareness to one's daily posture and movements through increased proprioceptive awareness is attained also through Yoga (Schmalzl et al., 2015). The increased proprioceptive awareness can help also to increase daily usage of desired components: Alexander Technique has also been demonstrated to facilitate lightness (Cohen et al., 2015), which can enhance feelings of happiness (Shafir et al., 2016).

## Laban movement analysis (LMA)

LMA classifies movement components in four main categories: Body, Effort, Space, and Shape (**Table 2**). Components from these categories change during movement in real time, making LMA particularly useful for observing and noting movement nuance and phrasing in everyday situations and in therapy (for a full description of LMA, see Fernandes, 2015). Its descriptive language identifies qualitative and quantitative aspects of movement with words such as light, strong, sudden, forward, sink, or retreat. It is relatively easy for people to understand, and familiar to most dance movement therapists (Dayanim et al., 2006; White, 2009). LMA has been used to describe movements associated with diverse topics, from personality and emotional state (Levy and Duke, 2003; Koch, 2007) to communicative gestures (Zhao, 2001) and rat behavior (Foroud and Pellis, 2003), and some of its components were successfully measured with a single accelerometer (Kikhia et al., 2014) and identified by a Kinect camera (Bernstein et al., 2015).

In Shafir et al. (2016), participants improvised moving different combinations of LMA components and rated which emotion they felt while moving. A logistic regression analysis of these ratings yielded sets of Laban movement components which elicited feeling the emotions of anger, fear, sadness, or happiness: Anger was elicited by strong, sudden, advancing, or direct movements. Fear was elicited by retreating, condensing, enclosing, binding, or moving back. Sadness was elicited by passive weight, bringing the arms to the upper body (chest, shoulder or face), sinking, or dropping the head. Happiness was elicited by jumping, rising, spreading, and free, light, upward, or rhythmic movements. (video examples in Supplementary Material, Supplementary Videos 1–8). In this paper, we theorize how these findings might be used to influence affective state through movement.

## How LMA theories can guide the use of movement components to influence emotions

As an augmented approach to psychotherapy, we suggest that understanding the movement components associated with each emotion, moving them and experiencing their effects during movement therapy, then integrating those components consciously into everyday life can expand motor vocabulary, open new possibilities of action and consequently emotional spectrum. Likewise, when desired and appropriate, the capacity to diminish the prevalence of certain components might provide a tool for self-regulation through weakening pre-set responses and their associated feelings. For this process of change to unfold smoothly we suggest three LMA theories to support a gradual process of transformation:

- The **developmental progression** of movement components suggests the order of introducing new elements for expanding movement range.
- Laban's theory of **affinities of Effort, Space and Shape** can facilitate motor learning of new components.
- Laban's theory of the **progressive shift between movement components** can structure the movement change so it is more organic and less abrupt.

The trained movement therapist can use these theories to attend to the client's movement and verbal cues for readiness to change movement components, observe subtle expression of emotion, share these observations with the client, and suggest how to use movement for resiliency in everyday life (for examples, see Tsachor, 2013).

## The developmental progression of body, effort, space & shape

The developmental progression of acquiring and mastering LMA movement components is laid out in published works by Eddy (Eddy, 1991, 2012, 2016), Amighi et al. (1999), Hackney (Hackney and Weeks, 2002), Tortora (2006), and Tsachor (2013) and shown in Table 1. This perspective suggests that components mastered in each stage of development underlie the motor skills of the next stage. Accordingly, introducing movements from earlier stages provides the support necessary to develop mastery of movements that develop in later stages. First published by Tsachor (2013), the table is revised here to outline a developmental order of the appearance of those components associated with basic emotions. Used in conjunction with findings from Shafir et al. (2016), the chart can point to the order in which to develop movement skills supporting resilient emotional responses.

**Table 1:** Developmental progression of acquiring Body, Effort, Space and Shape, highlighting LMA components found to be associated with each emotion.

**Tasks/Age these first develop**	**Body**	**Effort**	**Space**	**Shape**
0–3 months	**Breath flow** underlies Effort Flow & Shape Flow.	**Flow Effort**	**Inner Space:** developing the sense of space within one's own body.	**Shape Flow:** One's body changes shape to relate to one's needs: Growing/Shrinking
Sensing oneself in one's body	Sensing one's weight establishes one's sense of own body.	Freeing and Binding Flow enables control of one's movement.		
Self–regulation & Accept/reject (via Shape)	Yielding weight (Passive weight) underlies receiving support needed for bonding, and grounding which is needed later for supporting oneself.	**Pre Space Effort**: Mastering visual focal distance; attend/avert gaze.		Expanding: Grow (i.e., become convex) to open to pleasant sensations, or Condensing: Shrink (i.e., become concave) to close away from noxious stimuli.
Differentiate oneself as an individual	**Core/Distal**: Coordinating movement between one's core (sense of self) and distal limbs that interact with the world through flexion/extension.	**Pre Weight Effort**: managing the head weight begins self-regulation of feeding and attention to needs.	**Kinesphere:** beginning to establish reach-space in near to mid-reach.	
3–12 months	**Head/Tail:** mastering head-spine-body coordination (e.g., rolling).	**Space Effort:** Interest in *where* people/objects are.	**Horizontal Plane:** Explores objects and spatial environment.	**Widening** to trust, experience of outer/**Narrowing** to return to inner resources underlies Spreading/Enclosing
Establishing oneself in Space	Awareness of own body, self, others.	Gaze focuses attention helping development of Direct/Indirect movements.		
Bridging between oneself and the external world.	**Upper/lower, Homo-lateral, Cross-lateral** in horizontal plane: creep, crawl. Upper body organization.	Eye contact bridges to others.	**Kinesphere:** establishing Mid-reach (to arms-length without secure support of shape adaptation)	**Directional Movement** bridges to environment, relating by reaching out with **Spoking/Arcing** movements.
9–24 months Establishing one's own Weight:	**Upper/lower:** Standing, Stooping, Lower body strength. Vertical yield & push.	**Weight Effort**: Intention is the developmental task, expressed via modulating exertion of force in Strong/Light movements.	**Vertical Plane** Up/down established from sitting to pull up to stand.	**Lengthening/Shortening** underlies development of Rising/Sinking
Developing a sense of who one is and one's impact on others & environment.	**Homo-lateral**: Single-limb reach patterns. Climbing, Walking. **Cross-lateral** exploration in vertical plane begins with any twist & 1st steps	Sensing movement of own body weight, pushing oneself. Interest in *what* people & objects are, mass/weight of things.	**Kinesphere:** Far Reach to explore one's world and reach for what one wants.	**Postural adaptations in support of reach:** Shape begins to securely adapt to far-reach space. Moments of shaping in torso & limbs (hug).
18–36 monthsDeveloping one's ability to take action in time.	**Sagittal Shift**: Lower Body mastery. Rhythmic Locomotor patterns in Time: Run/stop, jump, spin.	**Time Effort**: Sudden/ Sustain. Speed, stop/start, acceleration/deceleration.	**Sagittal Plane** Forward/Backward	**Bulging/Hollowing** underlies development of Advancing/Retreating
Moving through Space	**Cross-Lateral** coordination in the sagittal plane develops.	Decision-making through Time Interest in *when* people & things will appear (sequencing of events).	Moving into Space beyond one's Kinesphere. Exploration of paths. Locomotor momentum is initially contained by parents, walls, and other surfaces. Begins transitions between planes	Shaping in posture and gesture: Integration continues of Spreading/Enclosing Rising/SinkingAdvancing/Retreating
**3–7 years** Interactive adaptation and complex responses.	**Integrating developmental patterns**. Mastering alternating developmental patterns smoothly.	**Integrating and Mastering Effort combinations:** Use of expressive states and Action drives to develop functional skills.	**3-Dimensional and cross-planar movement, transverse pathways**	**Posture/gesture mergers** Understanding and interacting with non-verbal cues.
Modulation of movement to serve own needs in response to environment & others.	Develops & playfully explores locomotion from crawling & walking to running and leaping through space.	Imaginary play. Development of personal movement signature	

*This chart is a modified version of the one published in Tsachor (2013) integrating information published by Tortora (2006) (p. 82–83), Amighi et al. (1999) and conversations with Martha Eddy*.

*Each stage reflects the new developing skills and assumes inclusion of all previous skills. Loman (personal communication, 2011) identifies the primary plane for motor development during the first year as horizontal, the primary plane for motor development during the second year as vertical and the primary plane for motor development during the third year of life as sagittal. The developmental progression first occurs in the order and ages in the table and may recapitulate throughout life. Sometimes, children move to the next developmental stage before fully integrating skills from previous stages (e.g., walking without crawling first). Going back to experience and practice the motor developmental tasks of earlier stages can profoundly change coordination of movement components that rely upon the earlier stages*.

Movement components associated with each emotion based on Shafir et al. (2016) are highlighted in the table with the following colors:

*Happiness=Light, Free, Jump, Up, Rising, Spreading, Rhythmic Phrasing; highlighted with #F58243*.

*Sadness= Passive Weight, Arms-to-upper-body, Sinking; highlighted with #6Ecddd*.

*Fear= Bound, Backward, Retreating, Condensing, Enclosing; highlighted with #67Bd45*.

*Anger= Strong, Sudden, Direct, Advancing; highlighted with Red*.

Using the developmental approach, a person may move primarily with Shape components from one stage (such as directional movement, first mastered at 6–12 months), but rarely move components from a later stage (e.g., spreading, advancing, or retreating, first mastered at 18–36 months). Understanding that when we don't progress beyond one stage, it may be because movement from the previous stage was not mastered, we might choose to first reinforce skills from the preceding stage, such as Shape Flow (first mastered at 0–3 months), by, for example, easily expanding and condensing with the rhythms of breathing. This may then facilitate our ability to change shape in the core, eventually permitting spreading and rising (components found to be associated with happiness) or retreating and enclosing (which were associated with fear) all mastered first at 18–36 months. Non-judgmental naming of the developmental movement progression can encourage learning new movement options, facilitating choice in responding to one's own particular challenges.

## Teaching affinities of effort, space and shape

LMA identifies four main categories of Movement: Body, Effort, Space, and Shape. The teaching of Effort, Space and Shape has been shown to be effective for changing stuck movement patterns (Bales, 2006; de Souza, 2016). Laban's theory of “Space Harmony” posits that moving in specific directions in Space naturally affines with specific Efforts (movement dynamics components, such as light, strong, sudden, sustained) and Shape components (changes in the body's configuration, such as sinking, rising, spreading, retreating). For example, reaching upward (in space) often lengthens the torso, which takes one into rising (a Shape component) and supports lightness (an Effort component), while downward flexion (in Space) may shorten the torso with sinking (a Shape component), and facilitates strength (an Effort component). Each row in Table 2 shows a set of affinities. These affinities, established from observation of “how the body and its limbs are able to execute certain dynamic nuances in movement toward certain areas in space better than toward others” (Laban and Ullmann, 1966 p.30) relate specific intention in Space to specific Effort and Shape components. Moving in the Space direction affined to a desired Effort or Shape component can make it easier to learn (Dell et al., 1977; Fernandes, 2015). Using the previous example: moving up may facilitate learning to rise and to move lightly (first row in Table 2). Since most components related to emotions found by Shafir et al. (2016) are Effort and Shape components, we can facilitate changes in Effort and Shape by using the affined directions in Space, which can be easier to move, thereby potentially affecting emotions.

**Table 2:** LMA Components, with affinities of Space, Shape and Effort presented in rows.

**Affinities between Space, Shape, and Effort**		
**Space**	**Shape**	**Effort**
Upwards	Rise	Light
Downwards	Sink	Strong
Side across	Enclose	Direct
Side open	Spread	Indirect/flexible
Backward	Retreat	Sudden
Forward	Advance	Sustain

*LMA's four main **categories** and their components are: **Body, Effort, Space, Shape***.

***Body**. This category identifies body segments, their coordination, and body actions*.

***Effort** describes eight dynamic, qualitative components of movement, reflecting a person's attitude toward movement. Direct/Indirect: Attention or attitude toward a chosen pathway; Strong/Light: the activation of one's weight as force to carry out one's intention, or not activated, i.e., passive weight; Sudden/Sustained, the element of decision expressed via acceleration/deceleration; and Bound/Free Flow, the attitude toward the movement's progression expressed via the degree of control or release of movement*.

***Space** describes where the movement goes in space, such as the directions up/down; side open/side across or forward/back*.

**Shape** describes how the body sculpts itself in space: Modes of Shape Change are:

Shape Flow: expanding or condensing; Directional Movement such as spoking or arcing;

*Shape Qualities: rise/sink, spread/enclose, advance/retreat; and Shaping/Carving*.

***Phrasing** describes how these movement components change intensity over time, such as increasing/decreasing or repeating rhythmically*.

*The spatial sequence presented in the left column of the table (upwards, downwards, side-across, side-open, backwards, forwards) is a basic motor sequence supportive of mobilizing a full range of Shape and Effort through their affinities in Space*.

People with *any* movement capacity can expand movement range via affined components: A sequence such as up (affined with rise and light), down (affined with sink and strong), etc. can be done with any body part, and at any size of reach space, so people can begin practicing the new components with skills already within their repertoire.

## Laban's theory of shift between movement components

In order to feel that moving a new component connects authentically to our emotions, the transition to new movement repertoire can be supported by continuing some ongoing, familiar components. During movement, Laban's “Space Harmony” theories organize the way in which change among components occurs, and therefore can serve as a template to structure movement changes in a progressive fashion (Bartenieff, 1972), so changes of expression and emotion will not feel too abrupt. When changing movement components, one common Space Harmony pattern is to (1) Maintain one component from the previous movement, to stabilize the experience, (2) Let go of another component then (3) Add a new (missing) component. This allows new movement to have continuity with the familiar previous movement as it branches into a new expression (Laban and Ullmann, 1966; Dell et al., 1977; Longstaff, 2000).

Shafir et al. (2016) found that 2–4 components were sufficient to bring about the experience of each emotion. Each component predicted only one emotion. Consequently, when a component is *not* present, the stimuli eliciting that emotion are reduced, making emotional shift easier. Thus, the principles of Space Harmony can support the process of change. For example, our movement behavior may be dominated by the qualities that Shafir et al. (2016) associated with fear: retreating, condensing, enclosing, binding, back. Recognizing that movement often expresses behavior which was adaptive, and when we believe these movements are pervasive, no longer adaptive, or more frequent than useful, we may wish to provide a structured method to gradually diminish the prevalence of some components which reinforce fear.

Based on Space Harmony structure, gradual movement change will be done through several small shifts such as:

Shift 1: *Maintain* retreating and backwards movement while gradually *letting go of* binding, and *adding* rhythmicity (a happy characteristic) such as rocking.

Shift 2: *Maintain* rhythmicity and backwards, *let go* of condensing and enclosing, and *add* some spreading.

Shift 3: *Maintain* spreading and rhythmicity, eventually releasing the back direction, moving sideward, rhythmically.

In this example, the mover is supported through gradual change, and can attend to emotions and movement impulses, so changes occur when the mover is authentically ready. This change may take place in one session, or gradually over time. In therapy, the individual's experience and clinician's judgment about the timing and readiness for each change are crucial to support self-efficacy, so each person learns to use movement to self-regulate emotions in tandem with psychological insight in therapy.

## Summary

The methods outlined here for expanding beyond the limits of one's habitual movement components to a fuller range can empower people to exit the grip of intransigent emotions into less emotionally-laden states, or enhance capacity and access to other emotions. They can be applied during individual therapy, group DMT when choosing movements to suggest or reflect back to the group, or become a self-effective by-product of yoga, Alexander Technique, Tai Chi or other mindful movement practices. Moreover, the methods described above have potential to support resiliency through attunement to one's own emotions. By learning the components associated with emotions, and tracking the experience of them in one's body, we can become more cognizant of signals sent “by our body” about emotions. For example, learning to notice slight condensing or binding in response to a situation may precede the conscious perception of “fear” and facilitate recognition of subtle emotional shifts. Such embodied emotional awareness can lead to conscious emotional resiliency.

Changes in movement components can be done through conscious movement responses ranging from full-bodied movement (e.g., during dance or yoga) to unobtrusive qualitative changes during daily activity e.g., lightening up, as in Alexander Technique (Cohen et al., 2015). Behavioral changes and motor learning both require time to integrate and assimilate, and the time required for a somatic movement approach to achieve emotional resiliency through changes in daily motor behavior warrants investigation. Further studies are also needed to determine effectiveness of this approach in specific disorders. Lastly, further study is also warranted to understand and define the role of therapists and teachers in these processes, as professional knowledge is needed to know when it is appropriate to introduce new motor components, in particular for people who may be in a vulnerable/difficult emotional state. We caution against simplification or use of the implied results by those without significant movement training.

## Conclusion

Movement is the way in which we humans adapt ourselves to our changing circumstances. We constantly re-configure our Shape and Effort, in order to respond to inner and outer stimuli (Bartenieff and Lewis, 1980). The fact that so much of our movement is voluntary provides an innate mechanism for self-regulation that can be nurtured and strengthened. As we expand choices in our own body's movement, we become empowered to respond to our world using movement as coping strategy adaptive to our needs. Over time, developing our capacity to make subtle movement choices can empower us with embodied skills for emotional resiliency.

## Author contributions

All authors have made an equal, substantial, direct and intellectual contribution to the work, and approved it for publication.

### Conflict of interest statement

RT teaches in programs affiliated with the Laban/Bartenieff Institute of Movement Studies. She does not have any commercial or financial relationships that could be construed as a potential conflict of interest. The authors declare that the research was conducted in the absence of any commercial or financial relationships that could be construed as a potential conflict of interest.

## Supplementary material

These video-clips show combinations of Laban movement components executed clearly with the entire body. In life, these components may appear as subtle movements, perhaps perceived as slight changes in posture or gestures.

The videos supplemented are:

Supplementary Video 1 Movement comprised of anger components Sudden Advance Direct.

Supplementary Video 2 Movement comprised of fear components Condense Enclose Bind Twist Back.

Supplementary Video 3 Movement comprised of sadness components Passive Weight and Sink.

Supplementary Video 4 Movement comprised of sadness components Sink and head drop.

Supplementary Video 5 Movement comprised of fear components Retreat Condense Enclose Back.

Supplementary Video 6 Movement comprised of happiness components Rhythmicity Up Rise Light.

Supplementary Video 7 Movement comprised of happiness components Rhythmic Jump Free Light.

Supplementary Video 8 Movement comprised of happiness components Rhythmic Spread Free Light.

## Abbreviations

DMT: Dance Movement Therapy
LMA: Laban Movement Analysis.
